# ELISA, protein immunoprecipitation and line blot assays for anti-TIF1-gamma autoantibody detection in cancer-associated dermatomyositis

**DOI:** 10.1093/rheumatology/keac288

**Published:** 2022-05-17

**Authors:** Sandra Selickaja, Angeles S Galindo-Feria, Lara Dani, Tsuneyo Mimori, Johan Rönnelid, Marie Holmqvist, Ingrid E Lundberg, Paulius Venalis

**Affiliations:** Department of Experimental, Preventive and Clinical Medicine, Center for Innovative Medicine; Department of Rheumatology, Vilnius University Hospital Santaros Clinics, Vilnius, Lithuania; Division of Rheumatology, Department of Medicine, Solna, Karolinska Institutet; Center for Molecular Medicine, Karolinska Institutet and Karolinska University Hospital Solna; Division of Rheumatology, Department of Medicine, Solna, Karolinska Institutet; Division of Rheumatology, Karolinska University Hospital, Stockholm, Sweden; Department of Rheumatology and Clinical Immunology, Kyoto University Graduate school of Medicine; Ijinkai Takeda General Hospital, Kyoto, Japan; Department Immunology, Genetics and Pathology, Uppsala University, Uppsala; Division of Rheumatology, Karolinska University Hospital, Stockholm, Sweden; Division of Clinical Epidemiology, Department of Medicine Solna, Karolinska Institutet; Division of Rheumatology, Department of Medicine, Solna, Karolinska Institutet; Center for Molecular Medicine, Karolinska Institutet and Karolinska University Hospital Solna; Division of Rheumatology, Karolinska University Hospital, Stockholm, Sweden; Division of Rheumatology, Karolinska University Hospital, Stockholm, Sweden; Division of Medicine, Section of Rheumatology, Danderyd University Hospital, Stockholm, Sweden

**Keywords:** Anti-TIF1-gamma, ELISA, line blot, immunoprecipitation, cancer-associated DM

## Abstract

**Objectives:**

Anti‐TIF1-gamma autoantibodies can be detected with immunoprecipitation (IP), line blot (LB) and ELISA. We compared assay performance in patients with DM and the potential of these assays to detect anti-TIF1-gamma positive cancer-associated DM (CADM).

**Methods:**

We included sera from 131 patients with DM followed at Karolinska University Hospital, Stockholm, Sweden and 82 healthy controls. Serum samples taken at DM diagnosis were tested for anti-TIF1-gamma autoantibodies with IP, two ELISAs (in-house and commercial) and LB. Cancer diagnosis and dates were obtained from the Swedish national cancer register. CADM was defined as a malignancy that developed within 3 years of DM diagnosis.

**Results:**

Anti-TIF1-gamma autoantibodies were detected in 19/101 (18.8%), 15/113 (13.2%), 34/131 (26%) and 45/131 (34.4%) of the patients with IP, LB, in-house and commercial ELISA, respectively. The anti-TIF1-gamma results from the in-house ELISA were confirmed with IP in 93 of 101 (92%) cases, κ = 0.76, with a commercial ELISA in 110 of 131 (84%) cases, κ = 0.63, and with LB in 101 of 113 (89.3%) cases, κ = 0.67. Anti-TIF1-gamma results with IP were confirmed with LB in 85 of 92 (92.4%) cases, κ = 0.73. For detecting CADM, the anti-TIF1-gamma in-house ELISA had a sensitivity of 58% and specificity of 86%, the commercial ELISA had a sensitivity of 63% and specificity of 82%, IP had a sensitivity of 52% and specificity of 92%, LB had a sensitivity of 40% and specificity of 96%.

**Conclusion:**

The two anti-TIF1-gamma ELISA assays had advantages both for autoantibody detection and to identify anti-TIF1-gamma-positive CADM.

Rheumatology key messages

- There is a good agreement among the analysed anti-TIF1-gamma assays performed in a DM cohort.
- Anti-TIF1-gamma detected by ELISAs are better predictors for cancer in DM patients compared with immunoprecipitation/line blot.
- The anti-TIF1-gamma ELISA may be used as a complement to other assays in clinical practice.

## Introduction

Immunoprecipitation (IP) is considered a gold standard for anti-TIF1-gamma autoantibody detection in serum. However, the use of IP is restricted to few research laboratories because it is labour intensive and time consuming. Also, this assay does not provide quantitative results [1]. New assays to detect anti-TIF1-gamma autoantibodies have been developed and several commercial assays are available, for example ELISA and line blot assays (LB) [2–4]. LB is a technically simpler assay compared with IP, and gives a semiquantitative result when evaluated with densitometry [1], ELISA is a quantitative assay [5] and easy to perform. However, the diagnostic sensitivity and specificity of these commercially available assays vary widely, as does their performance in relation to cancer diagnosis in patients with DM. Studies comparing LB versus IP have reported conflicting results regarding anti-TIF1-gamma autoantibody sensitivity/specificity with respect to cancer-associated DM (CADM) [1, 6, 7], and there are no studies reporting how well ELISA performs. Since ELISA can give quantitative results, an agreement between ELISA and IP would provide additional information of interest as not only the presence but also levels of anti-TIF1-gamma autoantibodies may be of importance to predict cancer risk or recurrence of cancer in patients with DM [8]. A reliable anti-TIF1-gamma autoantibody ELISA would greatly facilitate the detection of anti-TIF1-gamma autoantibodies and at the same time potentially be used to predict cancer risk by providing anti-TIF1-gamma autoantibody levels.

The aims of this study were: (i) to compare the performance of two ELISAs, IP and LB for detection of anti-TIF1-gamma autoantibodies in patients with DM, and (ii) to compare the potential of these assays to identify anti-TIF1-gamma-positive CADM.

## Methods

We included sera from 131 patients with definite DM followed at Karolinska University Hospital, Stockholm (Sweden) between 1996 and 2017 and 82 healthy controls (HC) [9]. Serum samples taken at the time of DM diagnosis were used for the analyses. Data on concomitant systemic CTDs and autoantibodies were collected from medical records. Cancer diagnosis and dates of cancer were obtained from the Swedish national cancer register. CADM was defined as a malignancy that developed within 3 years of a diagnosis of DM. The study was approved by the Swedish Ethical Review Authority. Participating patients and HC gave written informed consent to use their data and serum for research purposes.

### Anti-TIF1-gamma autoantibody assays

Serum samples from the same time point, stored at 80°C, were used for anti-TIF1-gamma autoantibody detection by ELISA, IP and LB. An in-house ELISA was performed using recombinant full-length human TIF1-gamma protein (OriGene, Rockville, MD, USA) which was diluted in carbonate buffer at 0.25 μg/ml, pH 9.6, and coated on 384-well high binding plates (Corning, Sigma-Arldrich, Burlington, MA, USA) overnight at 4°C. All other ELISA steps are the same as previously described [10]. A control serum sample was included as a standard curve in all plates to control for intra-plate variation. All samples were tested in duplicates. The results of the in-house ELISA were confirmed with a commercial ELISA kit (MBL, Japan) [11].

The LB assay (Euroline Myositis Antigen Profile 4, Euroimmun, Lübeck, Germany) with a densitometrical evaluation of staining intensity for 16 IgG autoantibodies was performed at the Department of Clinical Immunology, Uppsala University Hospital. The results were reported as negative [0–10 densitometry units (DU)], borderline (6–10 DU) or positive (≥11 DU) according to the manufacturer’s instructions. To compare sensitivities of ELISA and LB, the specificities were aligned by regarding borderline LB reactivities as positive and increasing the ELISA cut-off point until specificity rose to the level of LB. A Protein-IP assay was performed at Kyoto University as previously described [12].

### Statistics

A cut-off value for in-house ELISA was determined using a receiver operating characteristic curve with an area under the curve of 0.67 (37.7 AU/ml, with sensitivity 28.8%, specificity 97.6% and likelihood ratio 11.8). The cut-off value of the commercial ELISA was 7 U/ml as reported previously [11].

The sensitivity and specificity were evaluated, and McNemar’s test was used to compare the sensitivity and specificity between the assays. The Cohen kappa coefficient (κ) was used to evaluate the concordance between the assays. IBM SPSS Statistic v.20.0 was used for data management and statistical analyses. The results were considered statistically significant when *P* < 0.05.

## Results

Demographic data are presented in Table 1. The prevalence of CADM in the patient cohort was 29%.

**Table 1:** Demographic data of anti-TIF1-gamma-positive and -negative patients detected by in-house ELISA, IP and LB assays

	Total, *n* (%)	Mean age at cancer diagnosis, mean (s.d.)	Female, *n* (%)	Male, *n* (%)	Cancer, *n* (%)	CADM, %	Skin involve-ment, *n* (%)	Muscle involve-ment, *n* (%)	Other rheumatic diseases, *n* (%)	Other autoantibodies, *n* (%)
In-house ELISA anti-TIF1-gamma+	34 (26.0)	63.2 (12.4)	22 (64.7)	12 (35.3)	22 (64.7)	58	38 (100)	34 (100)	3 (8.8)	11 (32.3)
In-house ELISA anti-TIF1-gamma–	97 (74.0)	64.9 (10.6)	46 (47.4)	51 (52.6)	16 (16.5)	42	84 (86.6)	92 (94.8)	5 (5.1)	53 (54.6)
IP anti-TIF1-gamma+	19 (18.8)	63.9 (12.1)	13 (68.4)	6 (31.6)	14 (73.7)	52	18 (94.8)	17 (89.5)	2 (10.5)	7 (36.8)
IP anti-TIF1-gamma–	82 (81.2)	67.5 (9.8)	38 (46.3)	44 (53.7)	13 (15.6)	48	70 (85.3)	76 (92.7)	5 (6.0)	45 (54.9)
LB anti-TIF1-gamma+	15 (13.3)	64.1 (11.5)	12 (80.0)	3 (20.0)	12 (80.0)	40	14 (93.3)	14 (93.3)	2 (13.3)	6 (40.0)
LB anti-TIF1-gamma–	98 (86.7)	63.5 (14.1)	48 (49.0)	50 (51.0)	18 (18.4)	60	87 (88.8)	92 (93.4)	7 (7.1)	57 (58.1)

IP: immunoprecipitation; LB: line blot; CADM: cancer-associated DM.

Anti-TIF1-gamma autoantibody results were available for comparison between in-house ELISA and IP assay in 101 patients with DM. Twenty-four patients (24%) were anti-TIF1-gamma positive by ELISA, and 18 patients (18%) by IP.

Anti-TIF1-gamma autoantibody results were available for comparison between commercial and in-house ELISA in 131 patients with DM. Forty-five (34%) patients were anti-TIF1-gamma positive by commercial ELISA, and 38 (29%) by in-house ELISA.

For the comparison between in-house ELISA and LB, we had results from 113 patients. Twenty-eight patients (25%) were anti-TIF1-gamma positive by ELISA, and 18 patients (16%) by LB.

For comparison between IP and LB, there was available information from 92 patients. Eighteen patients (20%) were anti-TIF1-gamma positive by IP, and 14 patients (15%) by LB.

**Fig. 1:**
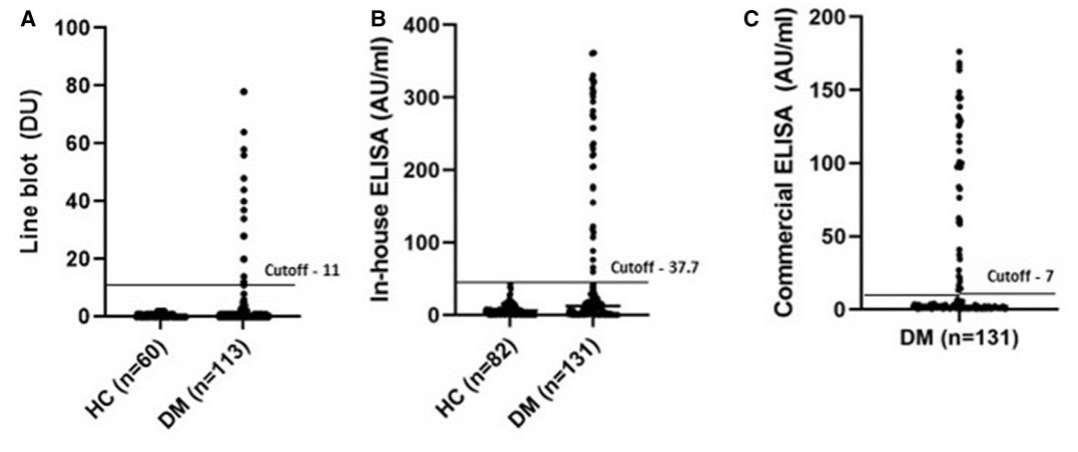
The distribution of anti-TIF1-gamma levels in the LB and ELISAs among HC and DM patients Panels (**A**), (**B**) and (**C**) demonstrate anti-TIF1-gamma autoantibody levels from a line blot (LB) assay (A) and in-house ELISA (B) among healthy controls (HC) and patients with DM, and anti-TIF1-gamma autoantibody levels from a commercial ELISA (C) among patients with DM. Each data point represents one individual and horizontal lines (black) indicate the mean values. DU: density units.

The distribution of anti-TIF1-gamma levels in the LB, and in the ELISAs among HC and DM patients is presented in Fig. 1.

### Comparison between the assays

The combined information on cancer and anti-TIF1-gamma autoantibody results were available for 127 patients analysed by ELISA, 101 by IP and for 113 by LB.

Firstly, we compared the presence of anti-TIF1-gamma autoantibodies detected by the in-house ELISA and the IP assay. Anti-TIF1-gamma results with ELISA were confirmed with IP in 93 of 101 (92%) cases, κ = 0.76. Discrepancies were found in eight cases. Six patients were anti-TIF1-gamma-positive by ELISA but negative by IP. Five of the six patients developed cancer within 3 years of DM diagnosis. Two serum samples were positive by the IP assay but negative by ELISA. These patients developed cancer within 1 year.

Secondly, we compared the results of the in-house ELISA with the commercial ELISA kit. Anti-TIF1-gamma results with the in-house ELISA were confirmed with the commercial ELISA in 110 of 131 (84%) cases, κ = 0.63. Discrepancies were found in 21 cases. Fourteen patients were anti-TIF1-gamma positive by commercial ELISA but negative by in-house ELISA, and three of these patients had cancer. Of the seven patients positive by in-house ELISA but negative by commercial ELISA, one had cancer.

Thirdly, we compared the in-house ELISA with LB assay. Anti-TIF1-gamma results with the in-house ELISA were confirmed with LB in 101 of 113 (89.3%) cases, κ = 0.67. Discrepancies were found in 12 cases. Eleven patients were positive by ELISA, and negative by LB. Eight of these 11 patients developed cancer within 3 years of DM diagnosis. One patient with cancer was anti-TIF1-gamma positive by LB but negative by ELISA.

Also, we compared the protein IP with the LB assay. Anti-TIF1-gamma results with IP were confirmed with LB in 85 of 92 (92.4%) cases, κ = 0.73. Discrepancies were found in seven cases. All patients were positive by IP but negative by LB, and five patients developed cancer within 3 years of DM diagnosis.

### The detection capability of different assays for cancer-associated DM

Next, we investigated the performance of the methods regarding the detection of cancer in patients with DM. The anti-TIF1-gamma in-house ELISA had a sensitivity of 58% and specificity of 86% for detecting CADM. The commercial ELISA had a sensitivity for CADM of 63% and specificity of 82%. Anti-TIF1-gamma autoantibodies identified by IP had a sensitivity of 52% and specificity of 92% to detect CADM. Anti-TIF1-gamma autoantibody results from the LB assay for detecting CADM had a sensitivity of 40% and specificity of 96%, which was a significantly lower sensitivity compared with ELISAs and IP (*P* < 0.05).

When we reduced the cut-off value for the LB assay to include borderline positives, we identified one additional patient who also had cancer. Even if we consider this patient as anti-TIF1-gamma positive, the sensitivity of the LB assay would only increase to 42%. When we adjusted the ELISA specificity to this LB specificity, the sensitivity for ELISA remained the same. The sensitivities and specificities of anti-TIF1-gamma autoantibody positivity in relation to CADM diagnosis by in-house ELISA, IP and LB assays are presented in supplementary Figs S1 and S2, available at *Rheumatology* online.

## Discussion

In our study, where identical serum samples were tested for detection of anti-TIF1-gamma autoantibodies using four different assays, we found an overall good concordance among them. Additionally, in association with cancer, the IP and ELISA assays were more sensitive compared with the LB assay.

A good agreement between the ELISA and IP assay for the detection of anti-TIF1-gamma autoantibodies has previously been reported [13]. The agreement between the IP and LB assays in our study was slightly better compared with some previous studies [4, 14] and slightly worse compared with another [1]. The observed differences may be explained by the different numbers of patients with DM included in each study. A strength of our study is the high number of patients with DM, the target group for anti-TIF1-gamma autoantibodies, and the valid information of cancer development over time.

Different explanations can be considered for the discrepant results between the assays. For the serum sample that was positive by IP, but negative by ELISA, a cross-reactivity in the IP cannot be excluded as the TIF1 antigen consists of two proteins (the 155 kDa gamma and the 140 kDa alpha chains), and the 155 kDa protein in the IP line can be difficult to distinguish from 150 kDa proteins, such as the Mi-2, leading to false-positive results [3]. On the other hand, some samples were positive by ELISA but negative by IP or LB. A false-positive ELISA cannot be ruled out in some cases. One explanation for false-positive ELISA could be cross-reactivity with autoantibodies found in other autoimmune diseases, such as SS [13]. It is also well known that detection of anti-TIF1-gamma by ELISA often encounters a problem of cross-reactivity between TIF1-gamma and Mi-2-beta [15]. Only a few of the patients who were positive by in-house ELISA but negative by IP or LB had known concomitant autoantibodies, however five patients who were positive by the commercial ELISA but negative by IP, LB or in-house ELISA had anti-Mi-2 autoantibodies which could interfere with the ELISA results. When we analysed the patients with divergent results between the methods, we found that almost half of in-house ELISA-positive and IP-negative patients had cancer, most of in-house ELISA-positive but LB-negative patients had cancer, and all IP-positive and LB-negative patients had cancer. In relation to cancer diagnosis, ELISA and IP demonstrated similar results with slightly higher sensitivity and slightly lower specificity of ELISA. However, the LB assay had a low sensitivity for anti-TIF1-gamma CADM detection compared with the other assays, as previously reported [16].

A strength of our study is that the same serum samples were used in the four autoantibody assays in a relatively large cohort of patients with DM. Another strength is that the information on cancer diagnosis was retrieved by linking the personal identification number to the national cancer registry, which includes almost 100% of cancer diagnoses of patients living in Sweden, indicating a high validity of the cancer diagnosis. This also made it possible to test the sensitivity and specificity for cancer associated with anti-TIF1-gamma autoantibodies up to 20 years from DM diagnosis. Also we had the possibility to confirm the in-house ELISA results with results obtained with a commercial ELISA. These analyses confirmed that detection of anti-TIF1-gamma autoantibodies with ELISAs or IP assays is superior to LB to predict cancer in patients with DM.

## Conclusions

We analysed the performance of four different assays to detect anti-TIF1-gamma autoantibodies and found a good agreement of the four tested assays to detect anti-TIF1-gamma autoantibodies in a cohort of patients with DM. ELISAs and IP had similar good sensitivity and specificity for anti-TIF1-gamma positive CADM, whereas LB had lower sensitivity. Therefore, the anti-TIF1-gamma ELISAs have advantages over the other two assays both for autoantibody detection and to detect anti-TIF1-gamma CADM, suggesting that this assay may be used as a complement to other assays in clinical practice.

## Supplementary Material

keac288_Supplementary_Data

## Data Availability

The dataset used and analysed during the current study is available from the corresponding author on reasonable request. Patient-level data will be anonymised and study documents will be redacted to protect the privacy of the patients.

## References

[1] Cavazzana I , Fredi M, Ceribelli A et al Testing for myositis specific autoantibodies: comparison between line blot and immunoprecipitation assays in 57 myositis sera. J Immunol Methods 2016;433:1–5. 26906088 10.1016/j.jim.2016.02.017

[2] Trallero-Araguas E , Rodrigo-Pendas JA, Selva-O’Callaghan A et al Usefulness of anti-p155 autoantibody for diagnosing cancer-associated dermatomyositis. A systematic review and meta-analysis. Arthritis Rheum 2012;64:523–32. 21953614 10.1002/art.33379

[3] Labrador-Horrillo M , Martínez MA, Selva-O’Callaghan A et al Anti-TIF1γ antibodies (anti-p155) in adult patients with dermatomyositis: comparison of different diagnostic assays. Ann Rheum Dis 2012;71:993–6. 22294626 10.1136/annrheumdis-2011-200871

[4] Espinosa-Ortega F , Holmqvist M, Alexanderson H et al Comparison of autoantibody specificities tested by a line blot assay and immunoprecipitation-based algorithm in patients with idiopathic inflammatory myopathies. Ann Rheum Dis 2019;78:858–60. 30760469 10.1136/annrheumdis-2018-214690

[5] Engvall E , Perlmann P. Enzyme-linked immunosorbent assay (ELISA). Quantitative assay of immunoglobulin G. Immunochemistry 1971;8:871–4. 5135623 10.1016/0019-2791(71)90454-x

[6] Montagnese F , Babačić H, Eichhorn P, Schoser B. Evaluating the diagnostic utility of new line immunoassays for myositis antibodies in clinical practice: a retrospective study. J Neurol 2019;266:1358–66.8. 30840145 10.1007/s00415-019-09266-4

[7] Cavazzana I , Richards M, Bentow C et al Evaluation of a novel particle-based assay for detection of autoantibodies in idiopathic inflammatory myopathies. J Immunol Methods 2019;474:112661. 31442464 10.1016/j.jim.2019.112661

[8] Ikeda N , Yamaguchi Y, Kanaoka M et al Clinical significance of serum levels of anti-transcriptional intermediary factor 1-c antibody in patients with dermatomyositis. J Dermatol 2020;47:490–6. 32103537 10.1111/1346-8138.15284

[9] Bohan A , Peter JB. Polymyositis and dermatomyositis (first of two parts). N Engl J Med 1975;292:344–7. 1090839 10.1056/NEJM197502132920706

[10] Venalis P , Selickaja S, Lundberg K, Rugiene R, Lundberg IE. Association of anti–transcription intermediary factor 1c antibodies with paraneoplastic rheumatic syndromes other than dermatomyositis. Arthritis Care Res (Hoboken) 2018;70:648–51. 28704599 10.1002/acr.23325

[11] Fiorentino DF , Gutierrez-Alamillo L, Hines D, Yang Q, Casciola-Rosen L. Distinct dermatomyositis populations are detected with different autoantibody assay platforms. Clin Exp Rheumatol 2019;37:1048–105. 31376258 PMC7039699

[12] Nakashima R , Imura Y, Kobayashi S et al The RIG-I-like receptor IFIH1/MDA5 is a dermatomyositis-specific autoantigen identified by the anti-CADM-140 antibody. Rheumatology (Oxford) 2010;49:433–40. 20015976 10.1093/rheumatology/kep375

[13] Aggarwal R , Oddis CV, Goudeau D et al Anti-transcription intermediary factor 1-gamma autoantibody ELISA development and validation. Rheumatology (Oxford) 2014;53:433–7. 24255164 10.1093/rheumatology/ket383 PMC3930887

[14] Tansley SL , Li D, Betteridge ZE, McHugh NJ. The reliability of immunoassays to detect autoantibodies in patients with myositis is dependent on autoantibody specificity. Rheumatology (Oxford) 2020;59:2109–14. 32030410 10.1093/rheumatology/keaa021 PMC7382594

[15] Fujimoto M , Murakami A, Kurei S et al Enzyme-linked immunosorbent assays for detection of anti-transcriptional intermediary factor-1 gamma and anti-Mi-2 autoantibodies in dermatomyositis. J Dermatol Sci 2016;84:272–81. 27693019 10.1016/j.jdermsci.2016.09.013

[16] Mariscal A , Milán M, Baucells A et al Anti-TIF-1γ antibody detection using a commercial kit vs in-house immunoblot: usefulness in clinical practice. Front Immunol 2021;11:625896. 33613568 10.3389/fimmu.2020.625896 PMC7894254

