# Supplementary material for: ELISA, protein immunoprecipitation and line blot assays for anti-TIF1-gamma autoantibody detection in cancer-associated dermatomyositis
Source: Rheumatology (Oxford). 2022 May 17;61(12):4991–6. doi: 10.1093/rheumatology/keac288 (PMC9707101; doi:10.1093/rheumatology/keac288)
Supplement: keac288_Supplementary_Data [file keac288_supplementary_data.docx]

**Supplementary Figure S1.** Anti-TIF1-gamma autoantibody results by in-house ELISA, IP, LB, and sensitivity when cancer occurred 1, 2, 3, 4, 5 years or at any time after dermatomyositis diagnosis.


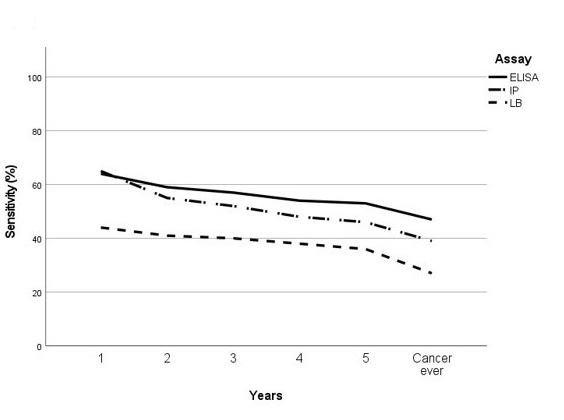


ELISA, Enzyme-linked immunosorbent assay; IP, Immunoprecipitation; LB, Line blot.

**Supplementary Figure S2.** Anti-TIF1-gamma autoantibody results by in-house ELISA, IP, LB, and specificity when cancer occurred 1, 2, 3, 4, 5 years or at any time after dermatomyositis diagnosis.


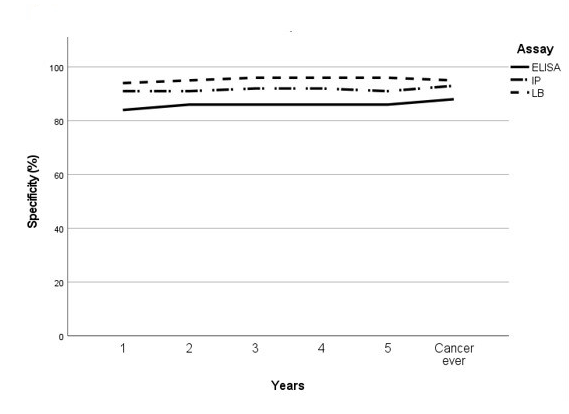


ELISA, Enzyme-linked immunosorbent assay; IP, Immunoprecipitation; LB, Line blot.

.
